# Functional recovery and mortality after intensive inpatient rehabilitation for severe acquired brain injury in older adults: a retrospective cohort study

**DOI:** 10.3389/fresc.2026.1901401

**Published:** 2026-09-03

**Authors:** Valeria Pingue, Chiara Pavese, Jasmine Invernizzi Descalzi, Loretta Fiorina, Vittorio Gabba, Gianluca Bellaviti, Antonio Nardone

**Affiliations:** 1 Department of Clinical-Surgical, Diagnostic and Pediatric Sciences, University of Pavia, Pavia, Italy; 2 Istituti Clinici Scientifici Maugeri IRCCS, Neurorehabilitation and Spinal Units and Centro Studi Attività Motorie (CSAM) of Pavia Institute, Pavia, Italy; 3 Clinical Laboratory, Microbiology and Virology Unit, Istituti Clinici Scientifici Maugeri IRCCS, Pavia, Italy; 4 Occupational Therapy and Ergonomics Unit of Pavia Institute, Istituti Clinici Scientifici Maugeri IRCCS, Pavia, Italy

**Keywords:** acquired brain injury, aging, functional outcome, neuro-rehabilitation, older adults

## Abstract

**Background:**

Severe acquired brain injury (sABI) is a major cause of mortality and long-term disability. Although its prevalence is increasing among older adults (≥65 years), evidence on independent determinant of rehabilitation outcomes in this population remains limited. This study aimed to identify early factors associated with poor functional outcomes and mortality in younger and older patients undergoing intensive inpatient rehabilitation following severe acquired brain injury (sABI).

**Methods:**

In this single-center, retrospective cohort study, 215 patients (91 aged ≥65; 124 aged <65) admitted to a tertiary neurorehabilitation unit were enrolled. Demographic characteristics, sABI etiology, comorbidities, dysphagia, and multidrug-resistant organism (MDRO) colonization at admission, as well as the occurrence of sepsis, hospital-acquired pneumonia (HAP), and in-hospital mortality, were evaluated. Multivariable linear regression models were used to identify factors independently associated with poorer outcomes in terms of Glasgow Coma Scale (GCS), Functional Independence Measure (FIM), and Glasgow Outcome Scale–Extended (GOS-E) scores recorded at discharge from the unit.

**Results:**

Among older patients, dysphagia (FIM *p* < 0.0001; GOS-E *p* = 0.022) and diabetes mellitus (FIM *p* = 0.006) were associated with poorer functional outcomes after sABI. In addition, MDRO colonization at admission (*p* = 0.016) and sepsis (*p* = 0.019) were associated with worse neurological status at discharge. In younger patients, dysphagia (FIM *p* < 0.0001; GOS-E *p* = 0.002) and MDRO colonization (FIM *p* = 0.007) were associated with poorer functional outcomes, whereas MDRO colonization at admission (*p* = 0.014) and HAP (*p* = 0.016) were associated with worse neurological status at discharge. Despite comparable functional outcomes at discharge, older patients had significantly longer LOS (*p* = 0.049) and higher in-hospital mortality (34 vs. 12, *p* < 0.0001). HAP was the factor most strongly associated with mortality in the overall cohort.

**Conclusions:**

Dysphagia and pre-existing comorbidities, particularly diabetes mellitus, were associated with poorer rehabilitation outcomes in older patients with sABI. The association of MDRO colonization, HAP, and sepsis with poor outcomes across both age groups underscores the importance of early identification of high-risk patients and targeted preventive strategies to reduce modifiable complications, length of stay, and mortality during intensive rehabilitation after sABI.

## Introduction

Severe acquired brain injury (sABI) refers to brain damage occurring after birth due to traumatic, or non-traumatic causes, including vascular, anoxic, infectious, metabolic, and neoplastic conditions (1).

Despite advances in acute care and therapeutic strategies, sABI remains a leading cause of mortality and long-term disability worldwide (2). Its management represents a major clinical challenge, often requiring prolonged, resource-intensive, multidisciplinary care pathways, including an intensive inpatient neurorehabilitation programme (2). Recent evidence indicates a rising prevalence of sABI among older adults (aged ≥65 years) across neurological registries (3, 4). This trend is largely driven by population ageing and improved survival after the acute phase of brain injury, regardless of its etiology (traumatic or non-traumatic) (5). In older adults, the increasing incidence of falls, which represent the leading cause of geriatric traumatic brain injury (TBI), contributes substantially to this phenomenon (6). Furthermore, ageing is associated with progressive cerebrovascular dysfunction, blood–brain barrier impairment (7), and a high burden of chronic comorbidities—including hypertension, diabetes, and atherosclerosis—which may increase susceptibility to cerebrovascular and anoxic brain injuries (8).

Most studies (3, 4, 6) investigating mortality-related factors and functional outcomes in patients with sABI identify chronological age as a major negative prognostic factor. Indeed, both injury mechanisms and the biological sequelae of sABI in older adults differ substantially from those observed in younger individuals (6). With ageing, cerebral white matter and the vascular system become more susceptible to injury, while endogenous reparative and neuroplastic mechanisms are progressively less effective (9). Moreover, in the geriatric population, functional outcomes and mortality are further influenced by pre-existing frailty and multiple comorbidities, which contribute to increased vulnerability and reduced potential of recovery (4). Unsurprisingly, these patients exhibit slower recovery trajectories and poorer functional and cognitive outcomes (3, 4, 6).

Despite the growing prevalence of sABI among older adults, evidence on the determinants of functional recovery in this population remains scarce. Most available studies have been conducted in acute or intensive care settings and focus predominantly on traumatic brain injury (TBI) (3, 4, 8, 10, 11), while evidence from the post-acute rehabilitation phase is scarce. As a result, the determinants of functional recovery in older patients with non-traumatic sABI remain poorly understood. Furthermore, older adults have historically been underrepresented in major TBI clinical trials, leaving clinicians with limited evidence-based guidance tailored to geriatric populations (10). This lack of data hinders the development of accurate prognostic models and personalized rehabilitation strategies for older patients with sABI.

This study aimed to identify factors independently associated with unfavourable functional outcomes at discharge among patients aged ≥65 years hospitalized for sABI and undergoing intensive inpatient rehabilitation after both traumatic and non-traumatic brain injury.

Secondarily, we conducted a survival analysis to compare mortality dynamics and risk between younger and older patients. These findings may help refine prognostic assessments and optimize clinical management in older adults, addressing an important gap in current neurorehabilitation research and potentially improving the allocation of hospital resources. Moreover, they may contribute to the development of specific prognostic models to identify patients most likely to benefit from intensive rehabilitation and targeted prevention strategies.

## Materials and methods

### Study design and cohort definition

In this single-center, retrospective cohort study, we consecutively enrolled patients admitted to the Neurorehabilitation Unit of the Istituti Clinici Scientifici (ICS) Maugeri in Pavia, Italy, a tertiary referral center dedicated to the post-acute management of sABI. The study design complied with the ethical guidelines of the Declaration of Helsinki and was approved by the local Ethics Committee of ICS Maugeri (ICS Maugeri, ref. 2214 CE, June 19, 2018). All participants, or their authorized representatives, signed a written informed consent.

Patients were eligible if they were admitted between December 1, 2019, and December 31, 2024, for care and rehabilitation following sABI. Inclusion criteria were: (1) age ≥18 years; and (2) admission to the neurorehabilitation unit within three months of sABI onset to continue clinical care and rehabilitation programs initiated in the Intensive Care Unit. Exclusion criteria were: (a) a history of pre-existing brain injury or other neurological diseases; and (b) incomplete or unavailable hospitalization data.

During the inpatient stay, all patients underwent an intensive, multidisciplinary rehabilitation program lasting up to six months, at least consisting of individualized 90-minute treatment sessions delivered six days per week.

### Variables, data sources

Data were extracted retrospectively and included: age at injury; sex; length of stay (LOS); sABI etiology; anamnestic comorbidities (including hypertension, diabetes mellitus, chronic kidney disease, ischemic heart disease, and hypothyroidism); presence of dysphagia at admission (clinically diagnosed by a speech therapist); use of invasive devices (e.g., tracheostomy, nasogastric tube, or percutaneous endoscopic gastrostomy); colonization with multidrug-resistant organisms (MDROs); occurrence of severe bacterial infections (hospital-acquired pneumonia and sepsis); and in-hospital mortality. Standardized data on dysphagia severity were not available. Anamnestic comorbidities, as well as other demographic and clinical variables, were extracted from electronic medical records, which systematically document patient information at admission and throughout hospitalization. Major infectious complications, particularly sepsis and hospital-acquired pneumonia (HAP), were identified from hospital discharge records.

### Definition of infectious disease

HAP was defined as any acute lower respiratory tract infection occurring at least 48 h after hospital admission (12), confirmed by a positive bacterial microbiological culture together with radiological evidence on chest radiography or computed tomography.

Sepsis was defined as life-threatening organ dysfunction caused by a dysregulated host response to infection. Organ dysfunction was identified as an acute increase in the total Sequential Organ Failure Assessment (SOFA) score of ≥2 points attributable to the infectious event (13).

Regarding colonization with MDROs, systematic screening on admission is routinely performed in our unit as part of the standard surveillance strategy. For the purposes of this study, we considered MDROs commonly implicated in nosocomial infections (14), including carbapenem-resistant *Enterobacterales*, carbapenem-resistant *Acinetobacter baumannii*, carbapenem-resistant *Pseudomonas aeruginosa*, vancomycin-resistant *Enterococcus faecium*, and methicillin-resistant *Staphylococcus aureus*.

### Neurological and functional assessment

The severity of brain injury was assessed using the Glasgow Coma Scale (GCS). The GCS is a widely used, standardized tool for evaluating the level of consciousness and the degree of neurological impairment across different types of brain injury (15). Total scores range from 3 to 15, with scores of 3–8 indicating severe brain injury, 9–12 moderate injury, and 13–15 mild injury (15).

Rehabilitation assessment was conducted on admission and discharge with the Functional Independence Measure (FIM) and the Glasgow Outcome Scale-extended (GOS-E). The FIM is a standardized instrument designed to assess and monitor progress in functional status through 13 motor (FIM-m) and 5 cognitive items (FIM-c) in terms of independence (14). Each of the 18 items is scored on a 7-point ordinal scale, yielding a total score ranging from 18 to 126, where 18 indicates complete dependence and 126 reflects complete functional independence (16).

The GOS-E is an eight-point ordinal scale that provides a global measure of functional outcome following brain injury, with particular reference to patients' level of dependence and degree of community reintegration. The scale ranges from 1 to 8, as follows: 1, death; 2, vegetative state; 3, lower severe disability; 4, upper severe disability; 5, lower moderate disability; 6, upper moderate disability; 7, lower good recovery; and 8, upper good recovery (17).

### Statistical analysis

Categorical variables were expressed as absolute numbers and percentages and were compared using the chi-square test. Continuous variables with a normal distribution were expressed as means and standard deviations and compared between the two age groups (≥65 years vs. <65 years) using the Student's t-test. Non-normally distributed continuous variables, including scale scores, were reported as medians and interquartile ranges (IQR) and compared using the Mann–Whitney U test.

Data were tested for normality of distribution using the Shapiro–Wilk test and, when appropriate, log-transformed to correct for skewness. Multivariate linear regression analyses were performed to investigate the potential role of clinical and demographic variables on functional outcomes in terms of FIM and GOS-E at discharge. The multivariate models included combinations of independent variables comprising sex (male = 0, female = 1), age (years), severity of brain injury as assessed by the GCS at admission, sABI etiology (traumatic = 1, non-traumatic = 0), presence of comorbidities, presence of invasive devices, dysphagia, colonization with MDROs, occurrence of HAP, and sepsis. We report the models with the highest adjusted R^2^ (Adj. R^2^) for each dependent variable. Kaplan–Meier analysis was used to estimate survival rates between the two age groups (≥65 years vs. <65 years). Differences in survival probabilities between groups were assessed using the log-rank test. Among the variables considered—sex, age, severity and etiology of brain injury, presence of comorbidities, dysphagia, and the occurrence of HAP and sepsis—multivariate logistic regression analysis was performed to identify potential factors associated with mortality. Odds ratio (OR), 95% confidence interval (95% CI), and related significant values obtained from the regression analysis were reported. Statistical analyses were performed using IBM SPSS Statistics version 21 (Somers, NY, USA). The threshold for statistical significance was set at *P* < 0.05.

## Results

### Clinical characteristics of the population

**Figure 1:**
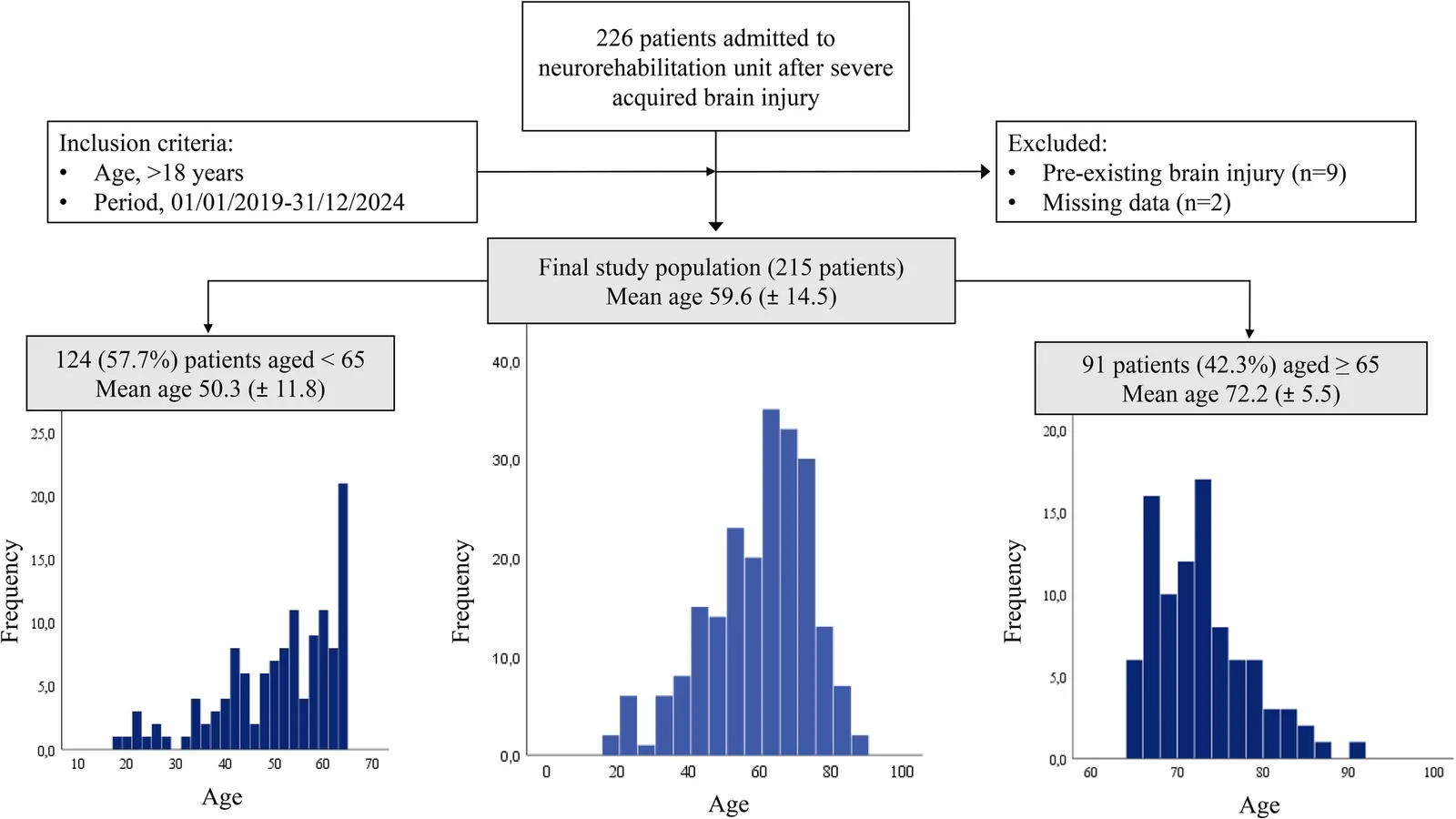
Patient selection process and distribution according to age group.

The cohort included 215 patients with a mean age on admission of 59.6 (±14.5) years (Figure 1).

Among these, 124 (57.7%, mean age 50.3 ± 11.8, standard deviation) were under 65 and 91 (42.3%, mean age 72.2 ± 5.5) were aged ≥65 years of age on admission.

Demographic and clinical characteristics of patients are presented in Table 1.

**Table 1:** Clinical and demographic characteristics of patients stratified by age group (≥65 years vs. <65)*.*

Variables		All patients *n* = 215	Age ≥65 *n* = 91 (42.3%)	Age <65 *n* = 124 (57.7%)	*p*
		*n* (%)	*n* (%)	*n* (%)	
Sex	Men	141 (65.6)	54 (59.3)	87 (70.1)	0.111
	Women	74 (34.4)	37 (40.7)	37 (29.8)	
Etiology	Traumatic	60 (27.9)	17 (18.7)	43 (34.7)	**0**.**013**
	Non-traumatic	155 (72.1)	74 (81.3)	81 (65.3)	
	Anoxic encephalopathy	21 (9.8)	10 (10.2)	11 (8.9)	0.647
	Cerebrovascular accident	118 (54.9)	60 (65.9)	58 (46.8)	**0**.**006**
	Primary brain tumor	16 (7.4)	4 (4.4)	12 (9.7)	0.191
Comorbidities	Hypertension	107 (49.8)	57 (62.4)	50 (40.3)	**0**.**001**
	Diabetes mellitus	37 (17.2)	22 (24.2)	15 (12.1)	**0**.**027**
	Chronic renal failure	12 (5.6)	7 (7.7)	5 (4.0)	0.367
	Ischemic cardiopathy	50 (23.2)	29 (31.9)	21 (16.9)	**0**.**014**
	Hypothyroidism	14 (6.5)	8 (8.8)	6 (4.8)	0.273
Colonization with MDROs on admission		140 (65.11)	58 (63.7)	82 (66.2)	0.772
Dysphagia on admission		185 (86.0)	80 (87.9)	105 (84.7)	0.554
Presence of device on admission	Tracheotomy	160 (74.1)	65 (71.4)	95 (76.6)	0.430
	Nasogastric tube	133 (61.9)	54 (59.3)	79 (63.7)	0.570
	PEG	54 (25.1)	23 (25.3)	31 (25.0)	0.875
Occurrence of infection	HAP	117 (54.4)	60 (65.9)	57 (45.9)	**0**.**005**
	Sepsis	68 (31.6)	21 (23.1)	47 (37.9)	**0**.**026**
Length of stay (days)		126 (56;198)	159 (64;202)	102 (48;187)	**0.049**
Death during hospitalization		46 (21.4)	34 (37.4)	12 (9.7)	**<0**.**0001**

Comparison between groups expressed as absolute number was performed by *χ*^2^ test or by Student's t test. Comparison between values, expressed as median and interquartile range, was performed by Mann–Whitney U test. Significant differences are in bold. MDROs, multidrug resistant organisms; PEG, percutaneous endoscopic gastrostomy; HAP, hospital acquired pneumonia.

The older patient group (aged ≥65 years) showed a significantly higher prevalence of non-traumatic etiology compared with younger patients (*p* = 0.013), particularly cerebrovascular accidents (*p* = 0.006). In this group, comorbidities at admission—including hypertension (*p* = 0.001), diabetes mellitus (*p* = 0.027), and ischemic cardiopathy (*p* = 0.014)—were significantly more frequent. HAP was significantly more frequent among patients aged ≥65 years (*p* = 0.005), whereas sepsis occurred more frequently in the younger patient group (*p* = 0.026). The median length of stay was 126 days (IQR 56–198), and older age was significantly associated with longer hospitalization (*p* = 0.049).

The comparison of the other baseline characteristics between the two groups (e.g., sex, presence of dysphagia or device or colonization by MDROs on admission) didn't show significant differences.

### Functional outcome

**Figure 2:**
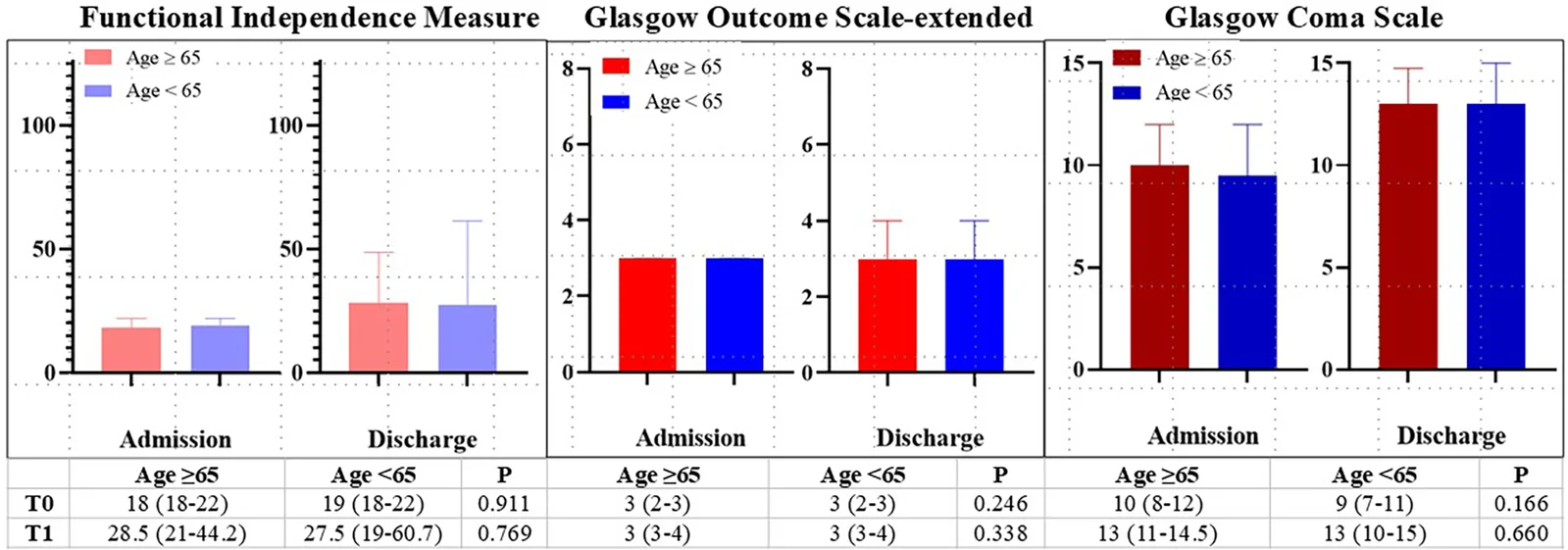
Comparison between age groups in neurological and functional assessments at admission and discharge from the rehabilitation unit. Values are reported as median (interquartile range), and between-group differences were assessed using the Mann–Whitney U test. Statistically significant results are highlighted in bold. GCS, Glasgow Coma Scale; FIM, functional independence measure; GOS-E, Glasgow Outcome Scale-Extended.

Figure 2 illustrates neurological and functional assessments at admission and discharge, as well as recovery during the rehabilitation period in both groups, with no significant differences observed between older and younger patients.

Table 2 shows the factors associated with neurological and functional outcomes in the two patient groups, as identified by multivariable linear regression analyses adjusted for age and sex.

**Table 2:** Multivariate linear regression analysis showing factors independently associated with neurological and functional outcomes at discharge (T1) in patients stratified by age.

Patients ≥ 65 years						
Independent variables	GCS T1 (Adj.R^2^ = 0.593)		FIM T1 (Adj.R^2^ = 0.801)		GOS-E T1 (Adj.R^2^ = 0.800)	
	*β*	*p*	*β*	*p*	*β*	*p*
Sex	−0.017	0.866	−0.039	0.559	−0.038	0.578
Age (years)	0.129	0.243	0.019	0.796	0.064	0.394
Etiology of injury	0.152	0.114	0.042	0.507	−0.020	0.751
GCS on admission	**0**.**553**	**0**.**001**	0.027	0.807	0.093	0.401
FIM on admission	−0.533	0.061	0.116	0.536	0.152	0.424
GOS-E on admission	0.607	0.053	0.240	0.245	**0**.**463**	**0**.**040**
Hypertension	0.049	0.639	−0.076	0.271	0.004	0.958
Diabetes Mellitus	−0.110	0.269	**−0**.**196**	**0**.**006**	−0.022	0.747
Chronic Renal Failure	−0.044	0.641	−0.076	0.246	0.033	0.611
Ischemic cardiopathy	0.073	0.508	0.128	0.092	0.018	0.804
Hypothyroidism	−0.069	0.479	−0.048	0.460	−0.051	0.439
Dysphagia on admission	0.035	0.827	**−0**.**555**	**<0**.**0001**	**−0**.**253**	**0**.**022**
Colonization by MDROs	**−0**.**409**	**0**.**016**	−0.190	0.069	−0.115	0.219
HAP	−0.149	0.172	0.003	0.969	0.047	0.519
Sepsis	**−0**.**212**	**0**.**019**	−0.051	0.438	−0.108	0.108

Patients < 65 years						
Independent variables	GCS (Adj.R^2^ = 0.445)		FIM (Adj.R^2^ = 0.593)		GOS-E (Adj.R^2^ = 0.479)	
	*β*	*p*	*β*	*p*	*β*	*p*
Sex	−0.076	0.365	0.084	0.275	**0**.**176**	**0**.**032**
Age (years)	−0.058	0.543	0.055	0.478	−0.095	0.318
Etiology of injury	0.043	0.629	0.059	0.465	0.144	0.110
GCS on admission	**0**.**447**	**0**.**000**	0.198	0.083	**0**.**301**	**0**.**012**
FIM on admission	0.014	0.885	**0**.**232**	**0**.**012**	0.149	0.139
GOS-E on admission	0.005	0.971	−0.050	0.684	0.037	0.786
Hypertension	0.017	0.843	0.045	0.556	0.054	0.521
Diabetes Mellitus	−0.050	0.538	−0.057	0.437	−0.047	0.558
Chronic Renal Failure	−0.009	0.906	−0.098	0.161	−0.013	0.861
Ischemic cardiopathy	0.002	0.975	−0.122	0.094	−0.017	0.834
Hypothyroidism	−0.043	0.569	−0.013	0.849	0.078	0.307
Dysphagia on admission	−0.147	0.127	**−0**.**418**	**<0**.**0001**	**−0**.**321**	**0**.**002**
Colonization by MDROs	**−0**.**250**	**0**.**014**	**−0**.**242**	**0**.**007**	−0.194	0.060
HAP	**−0**.**216**	**0**.**016**	−0.106	0.200	0.038	0.671
Sepsis	−0.143	0.107	−0.024	0.768	−0.125	0.162

Significant associations are reported in bold. GCS, Glasgow Coma Scale, MDROs, multidrug-resistant organisms; FIM, functional independence measure; GOS-E, Glasgow Outcome Scale-extended; MDROs, multidrug resistant organisms; HAP, hospital-acquired pneumonia.

The occurrence of sepsis in the older group (*β* = −0.212, *p* = 0.019) and HAP in the younger group (*β* = −0.216, *p* = 0.016) was associated with worse neurological assessment at discharge. Additionally, a better neurological status at admission (≥65 years: *β* = 0.553, *p* = 0.001; <65 years: *β* = 0.445, *p* < 0.0001) and the absence of MDRO colonization (≥65 years: *β* = −0.409, *p* = 0.016; <65 years: *β* = −0.250, *p* = 0.014) were independently associated with more favourable neurological outcomes in both age groups.

In the older patient group, the presence of dysphagia was the factor most strongly associated with worse functional outcome in terms of FIM (*β* = −0.555, *p* < 0.0001) and GOS-E (*β* = −0.253, *p* = 0.022) on discharge. Diabetes mellitus was also independently associated with worse functional outcomes, particularly in terms of FIM scores (*β* = −0.196, *p* = 0.006). In addition, better functional status at admission was significantly associated with more favorable rehabilitation outcomes at discharge, as reflected by higher GOS-E scores (*β* = 0.463, *p* = 0.040).

In the group of patients aged <65 years, the primary factors associated with poorer functional outcomes were dysphagia (FIM *β* = −0.418, *p* < 0.0001; GOS-E *β* = −0.321, *p* = 0.002), colonization by MDROs (FIM *β* = −0.242, *p* = 0.007), and male sex (GOS-E *β* = 0.176, *p* = 0.037). Finally, higher FIM and GOS-E scores on admission were significantly associated with better functional outcomes at discharge, as measured by the same tools.

### Mortality

**Figure 3:**
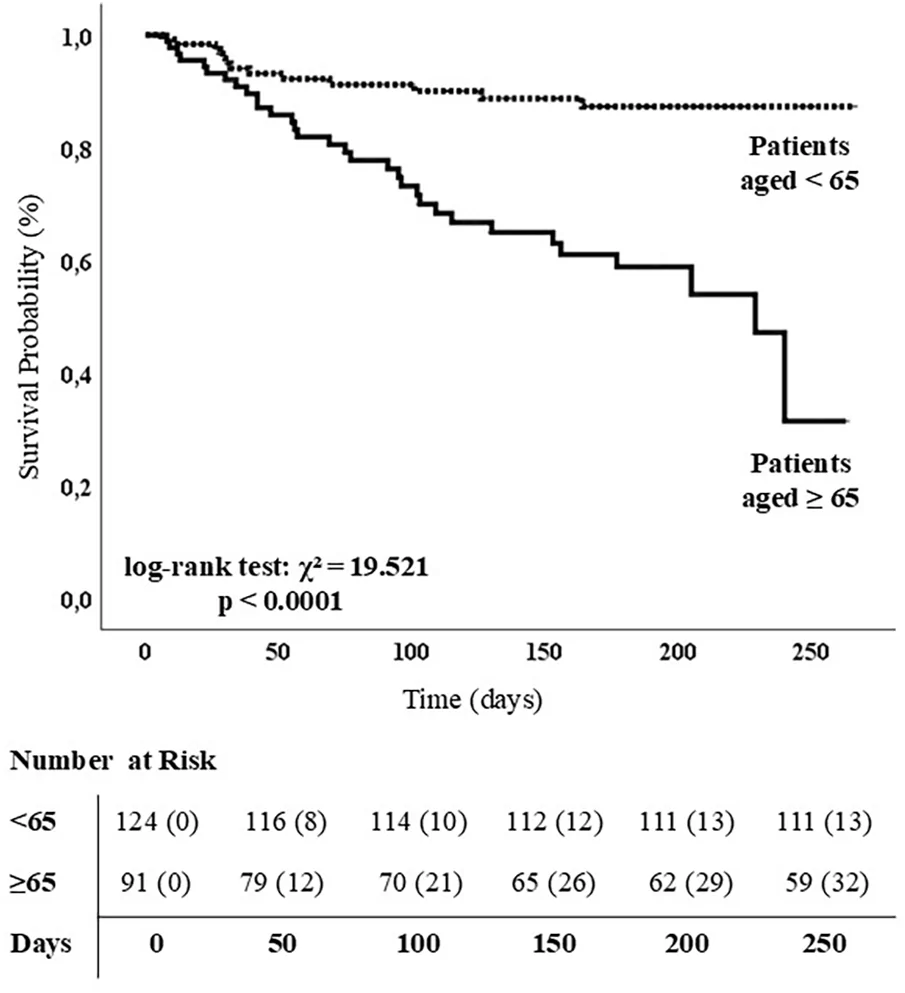
Kaplan–Meier survival analysis with number-at-risk table for the overall cohort, stratified by age (<65 years vs. ≥ 65 years).

Death during inpatient rehabilitation was documented in 46 (21.4%) patients after sABI. Mortality was significantly higher among patients aged ≥65 years (*n* = 34) compared with those aged <65 years (*n* = 12; *p* < 0.0001) (Table 1). Figure 3 shows the Kaplan–Meier survival curves for the two age groups, illustrating differences in survival probability over time.

The survival rate was significantly higher in patients aged <65 years compared with those aged ≥65 years (log-rank test: *χ*^2^ = 19.521; *p* < 0.0001). Notably, among older patients, the probability of survival dropped below 85% after approximately 250 days of hospitalization.

Finally, multivariate logistic regression analyses were performed to evaluate the potential contribution of the considered variables to mortality during inpatient rehabilitation in all the study population (Table 3).

**Table 3:** Factors associated with mortality during inpatient rehabilitation after severe acquired brain injury.

Covariates	OR (95% CI)	*p*
Age (≥ 65 years = 0; < 65 years = 1)	**0.111** (**0.021-0.562)**	**0**.**010**
Sex	1.511 (0.686–3.329)	0.306
Etiology of injury (traumatic = 1, non-traumatic = 0)	**0.246** (**0.076–0.797)**	**0**.**019**
Glasgow coma scale on admission	**0.851** (**0.740–0.978)**	**0**.**023**
Dysphagia on admission	2.604 (0.279–24.312)	0.401
Hospital acquired pneumonia	**2.923** (**1.190–7.180)**	**0**.**019**
Sepsis	0.900 (0.399–2.029)	0.800

Significant associations are reported in bold.

In the overall cohort, the occurrence of HAP emerged as the factor most strongly associated with mortality (*p* = 0.019). Specifically, the onset of HAP during hospitalization was independently associated with an almost threefold increase in mortality risk. In contrast, age <65 years (*p* = 0.010), traumatic etiology (*p* = 0.019), and higher neurological status at baseline (*p* = 0.023) were significantly associated with a lower risk of mortality.

## Discussion

In this study, we found that, among patients aged ≥65 years, the presence of dysphagia and diabetes mellitus was associated with poorer functional outcomes after sABI. In contrast, MDRO colonization on admission and the occurrence of sepsis during hospitalization were associated with worse neurological outcomes at discharge.

In the overall cohort, the occurrence of HAP showed the strongest independent association with mortality (*p* = 0.019). In this group, neurological outcomes were likewise negatively associated with the occurrence of infectious events during hospitalization and with MDRO colonization at admission.

sABI frequently leads to impaired physiological swallowing. The incidence of dysphagia has been reported to range from 8.1% to 80% among patients with stroke and from 27% to 30% in those with traumatic brain injury (18). Moreover, the prevalence of dysphagia increases with advancing age (19). In our cohort, dysphagia was highly prevalent on admission in both older (87.9%) and younger patients (84.7%), significantly impacting rehabilitation outcomes in both populations. These findings suggest that swallowing disorders reflect the severity of neurological injury independently of age. Notably, a recent retrospective study conducted in a small cohort of older patients with sABI (20) identified PEG placement for dysphagia as a significant marker of poor outcome. Indeed, dysphagia following sABI has been consistently associated with an increased risk of pneumonia (2) and malnutrition (19–21), conditions that may adversely influence rehabilitation trajectories and overall outcomes regardless of patient age (22).

In line with previous studies (23, 24), our data highlight that pre-existing comorbidity are key factor related with poor outcome in older patients after sABI. In particular, in our cohort, diabetes mellitus was significantly associated with worse outcomes. This finding may be explained by the systemic effects of diabetes, including chronic inflammation, microvascular damage, and metabolic dysregulation, all of which can impair neural recovery and muscle function (25). In addition, diabetes is associated with increased susceptibility to infections, further contributing to poorer outcomes (25).

Another factor associated with worse neurological outcomes in both groups, and with poorer functional outcomes among younger patients, was MDRO colonization. While MDRO colonization is a well-recognized risk factor for infection (14) and mortality in critical care settings (26), evidence regarding the relative impact on rehabilitation outcome after sABI remains limited. In the rehabilitation setting, this association may be partially explained by the increased risk of infectious complications and by reduced access to gyms due to infection-control measures, both of which can substantially affect functional recovery.

Regarding in-hospital mortality, older patients experienced higher mortality rates, largely driven by the occurrence of HAP, which emerged as the factor most strongly associated with mortality after sABI, consistent with previous findings (2). Patients with severe acquired brain injury (sABI) are known to have increased susceptibility to systemic infections due to clinical complexity and injury-induced immunosuppression (27). While these complications may reflect the severity of the primary neurological injury, they are potentially modifiable factors that, unlike injury severity itself, may independently increase the risk of mortality regardless of age. This may explain the strong association observed between infectious complications, particularly HAP and sepsis, and adverse outcomes in our cohort.

In contrast, younger age (<65 years), traumatic etiology, and better neurological status at admission were independently associated with a lower risk of mortality in the overall study cohort.

Interestingly, in our cohort, survivors aged ≥65 years exhibited a recovery trajectory comparable to that of younger patients, despite requiring a longer inpatient rehabilitation stay. These findings highlight the importance of early identification of high-risk patients, timely management of dysphagia, prevention of infectious complications, and individualized rehabilitation planning, to optimize recovery trajectories and potentially reduce LOS and mortality in this vulnerable population.

### Study limitations

This study has several limitations. First, its retrospective single-center design may limit the generalizability of the findings. Second, because data were extracted from electronic medical records, some potentially relevant clinical and functional variables were unavailable for analysis. For instance, although dysphagia was clinically assessed and diagnosed by a speech therapist, standardized information on its severity was not available. Consequently, dysphagia severity could not be directly measured and was only indirectly inferred from the presence of enteral feeding devices, such as a nasogastric tube or PEG. Moreover, several variables identified in this study, including dysphagia, invasive devices, hospital-acquired pneumonia, and sepsis, may reflect overall clinical severity rather than being independently associated with outcomes. To mitigate this potential confounding, multivariable analyses were adjusted for brain injury severity and other relevant baseline characteristics.

Nevertheless, the study also has important strengths. It included a relatively large cohort for this specific clinical population and focused on older adults undergoing intensive inpatient rehabilitation, a patient population and care setting that have been insufficiently investigated in previous studies.

### Conclusions and clinical implications

In summary, dysphagia and diabetes mellitus emerge as factors potentially associated with poorer rehabilitation outcomes in older adults following sABI. Moreover, our data underscore the crucial role of MDRO colonization and infectious events during intensive rehabilitation in contributing to worse neurological outcomes and increased mortality, regardless age of patients. Further studies, particularly prospective investigations, are needed to elucidate the mechanisms underlying these associations.

## Data Availability

The raw data supporting the conclusions of this article will be made available by the authors, without undue reservation.
